# A Case Report of Malignant Mesenchymal Tumor Presenting As Anterior Thigh Swelling: A Diagnostic Challenge

**DOI:** 10.7759/cureus.55669

**Published:** 2024-03-06

**Authors:** Jayashree Rey, Samarth Shukla, Sourya Acharya, Pravin Gadkari, Sapna Sihag

**Affiliations:** 1 Pathology, Jawaharlal Nehru Medical College, Datta Meghe Institute of Higher Education and Research, Wardha, IND; 2 Medicine, Jawaharlal Nehru Medical College, Datta Meghe Institute of Higher Education and Research, Wardha, IND; 3 Biochemistry, Dr. Sampurnanand Medical College, Jodhpur, IND

**Keywords:** multidisciplinary management, histopathological confirmation, soft tissue sarcoma, diagnostic challenge, anterior thigh swelling, malignant mesenchymal tumor

## Abstract

This case report describes the presentation, diagnostic challenges, and management of a 64-year-old male with a malignant mesenchymal tumor presenting as a swelling in the anterior thigh. Despite initial misdiagnosis and treatment at a local hospital, the swelling worsened, leading to referral to a specialized hospital. Further investigations, including blood tests, ultrasonography (USG), and MRI, revealed a large solid cystic lesion compressing adjacent muscles, indicative of soft tissue sarcoma (STS). A skin biopsy confirmed the diagnosis of a malignant mesenchymal tumor. The patient, also suffering from hypertension and diabetes mellitus, was subsequently referred to the oncology department for further management. This case underscores the importance of thorough evaluation and histopathological confirmation for accurate diagnosis and management of STS, particularly in the context of atypical presentations and comorbidities.

## Introduction

Soft tissue sarcomas (STS) represent a heterogeneous group of rare malignant tumors from mesenchymal cells, encompassing over 50 histological subtypes [1]. While they constitute less than 1% of all adult malignancies, STS can occur at any anatomical site and present with diverse clinical manifestations, posing diagnostic challenges [2].

The diagnosis of STS relies on a combination of clinical evaluation, imaging modalities, and histopathological analysis [3]. Imaging techniques such as ultrasonography (USG) and magnetic resonance imaging (MRI) play crucial roles in assessing tumor size, extent, and involvement of adjacent structures [4]. However, definitive diagnosis often requires histopathological confirmation through biopsy, which guides treatment planning and prognostication [5].

Treatment of STS typically involves a multidisciplinary approach, incorporating surgery, radiotherapy, and chemotherapy depending on tumor characteristics and patient factors [6]. Despite advancements in treatment modalities, the prognosis of STS remains variable, influenced by factors such as tumor histology, grade, size, and patient comorbidities [7].

## Case presentation

A 64-year-old male presented to the outpatient department of the specialized hospital in Wardha district, reporting swelling in the front area of his left thigh for the past 20 days. During the history-taking, the patient disclosed that the swelling had gradually developed without any apparent trigger, and despite receiving treatment at a local private hospital two weeks prior, the swelling had worsened. This prompted a referral to the super-specialty hospital. Additionally, the patient mentioned a history of bypass surgery in Nagpur 2.5 years ago, as well as a diagnosis of hypertension and diabetes mellitus for the past 1.5 years, for which he was taking Telmisartan 40 mg and Metformin 500 mg twice daily.

**Figure 1:**
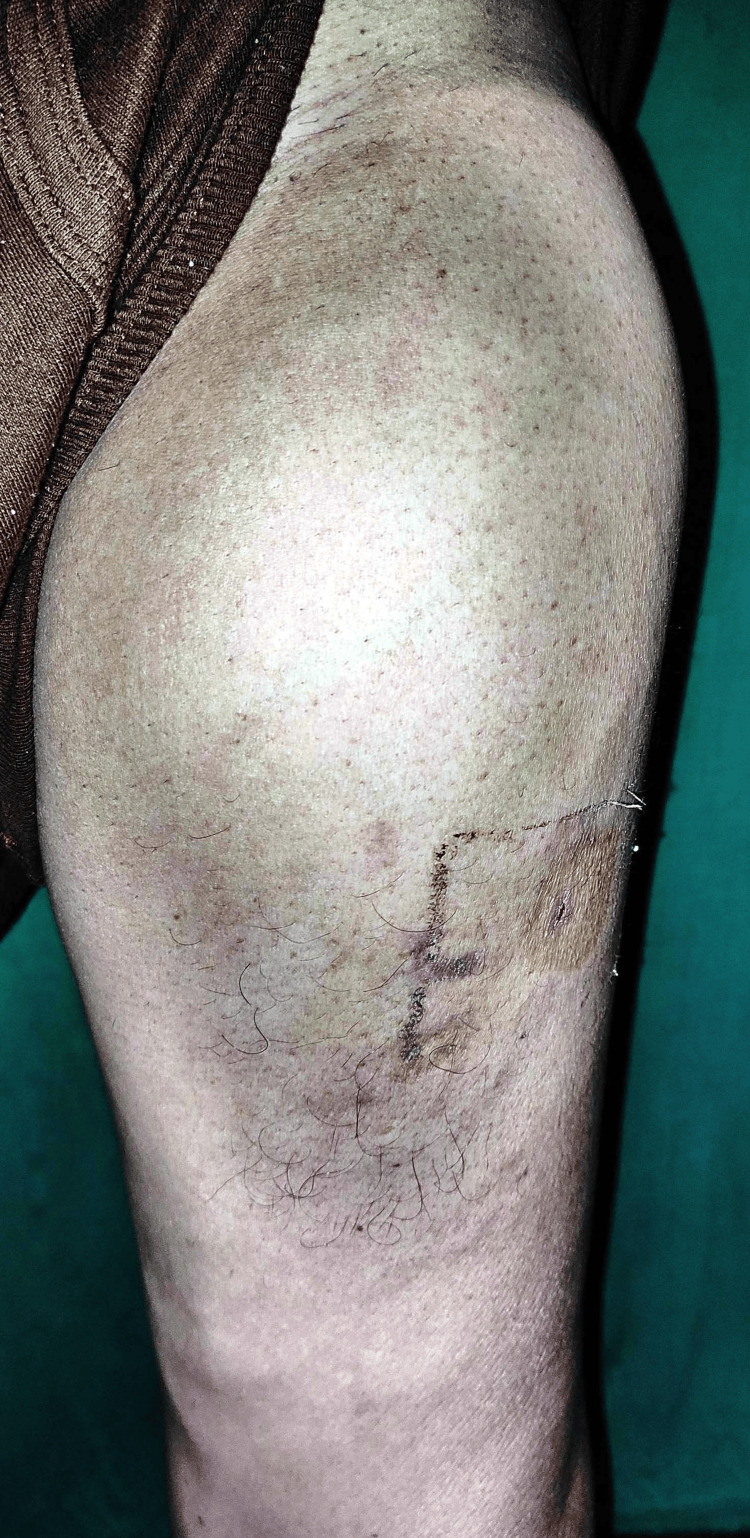
Shows an immobile lump with a temperature increase but no active discharge

Upon local examination, the medical team observed swelling and tenderness in the front area of the left thigh, initially measuring 2x1 cm and currently enlarged to 10x8 cm. Palpation revealed a soft, immobile lump with a temperature increase but no active discharge (Figure 1). Blood tests were recommended (Table 1).

**Table 1:** Laboratory investigation Hb, hemoglobin; RBC, red blood cell; MCV, mean corpuscular volume; MCH, mean cell hemoglobin; MCHC, mean corpuscular hemoglobin concentration; RDW, red cell distribution width; TLC, total leukocyte count

Name of Test	Patient Value	Reference Value
Hb	11.4	11-15 mg/dL
Glucose plasma random (RBS)	96	140-199 mg/dL
RBC	4.74	3.8-5.8 per mcL
MCV	72.4	76-96 fL
MCH	23.9	27-32 pg
MCHC	33	31-35 gm/dL
Platelet count	258000	150,000 to 400,000 per mcL
RDW	18.1	1-15%
TLC	10100	4000-11,000/mcL
Differential leukocyte count		
Granulocytes	75	40-75%
Lymphocytes	20	20-45%
Eosinophils	02	1-6%
Monocytes	03	2-10%
Basophils	00	0-1%
Kidney function test		
Serum sodium	137	135-145 mmol/L
Serum potassium	3.8	3.5-5.5 mmol/L
Serum urea	22	8 and 24 mg/dL
Serum creatinine	0.8	0.74 to 1.35 mg/dL
Liver function test		
Alkaline phosphatase	79	30-130 IU/L
ALT (SGPT)	19	7-56 U/L
AST (SGOT)	20	8-48 U/L
Total protein	7.2	6.0-8.3 g/dL
Albumin	3.7	3.5-5.5 g/dL
Total bilirubin	0.8	0.2-0.8 mg/dL
Bilirubin conjugated	0.3	0-0.2 mg/dL
Bilirubin unconjugated	0.5	0.2-0.6 mg/dL

**Figure 2:**
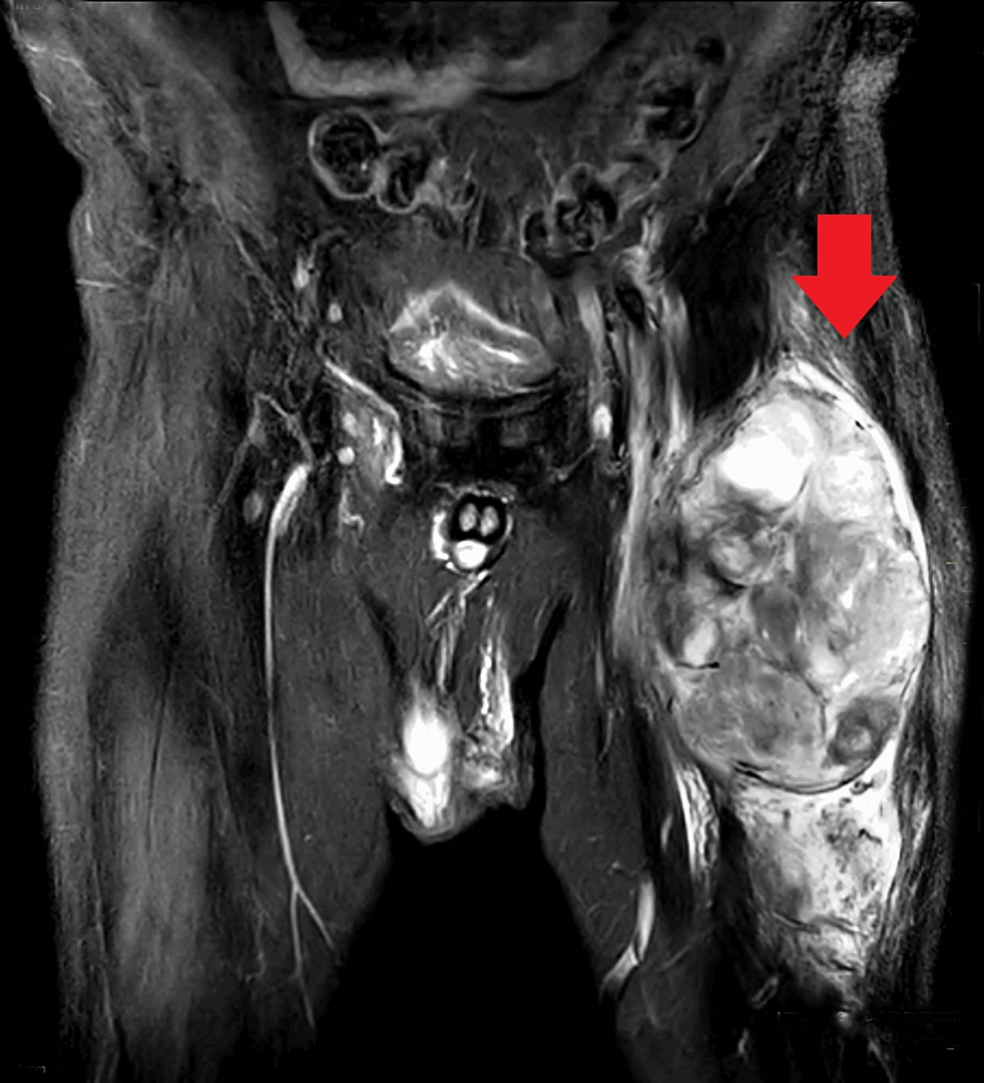
Shows large ovoid mixed solid cystic lesion in the anterolateral aspect of the left thigh, causing displacement of adjacent muscles but no infiltration into surrounding tissues

Further investigations included USG of the left thigh, revealing a well-defined encapsulated solid cystic heterogeneity hypercellular lesion compressing underlying muscles. Minimal internal vascularity suggested a benign nature. MRI with contrast confirmed a large ovoid mixed solid cystic lesion in the anterolateral aspect of the left thigh, causing displacement of adjacent muscles but no infiltration into surrounding tissues (Figure 2). Subchondral cysts were noted in the right femur. These findings were indicative of STS and neurogenic tumors.

**Figure 3:**
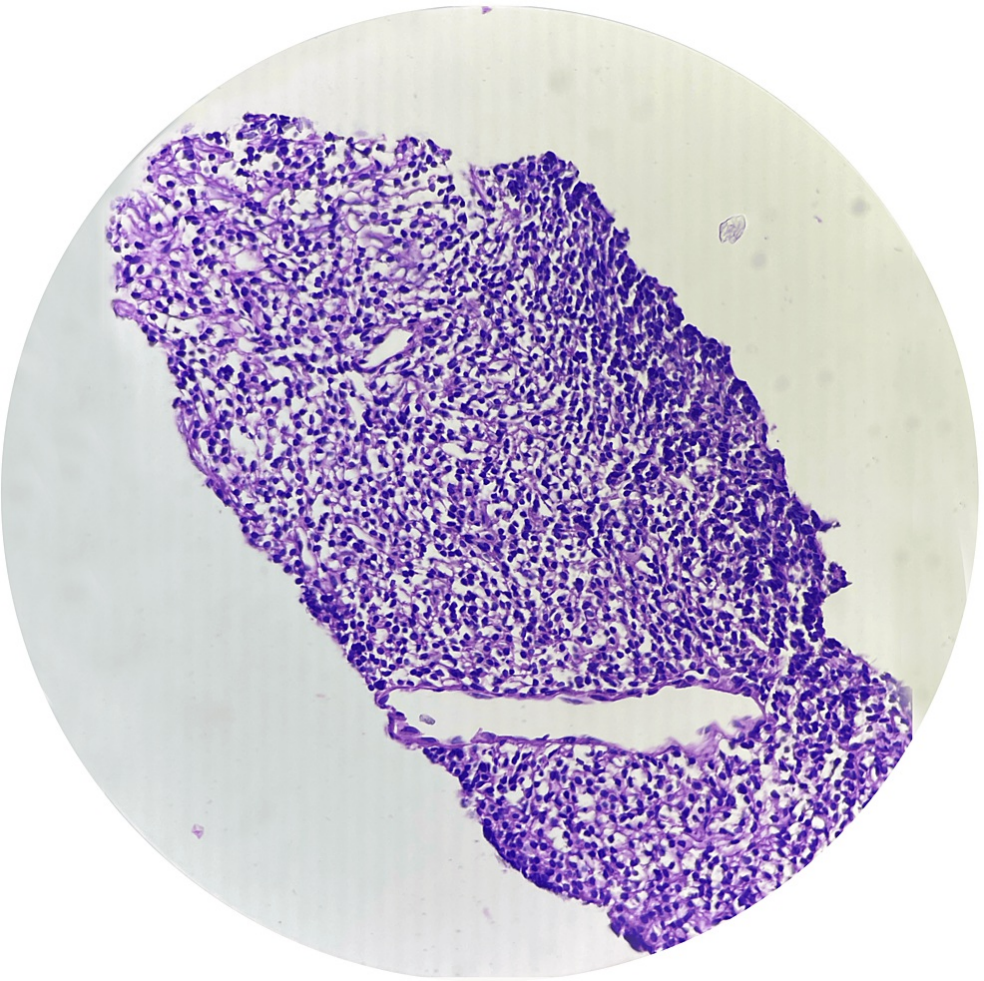
Shows malignant mesenchymal tumor (40×) Hematoxylin and eosin (H&E) was used.

A soft tissue biopsy confirmed the diagnosis of a malignant mesenchymal tumor. Consequently, the patient was diagnosed with a malignant mesenchymal tumor in the context of hypertension and diabetes mellitus and was referred to the oncology department for further treatment (Figure 3).

## Discussion

STS represent a challenging group of tumors due to their diverse histological subtypes and variable clinical presentations. This case underscores the importance of a comprehensive diagnostic approach and multidisciplinary management in a malignant mesenchymal tumor presenting as an anterior thigh swelling. The initial presentation of the patient with a gradually enlarging thigh swelling highlights the importance of considering malignant etiologies in the differential diagnosis, particularly in older individuals with risk factors such as prior surgeries and comorbidities like hypertension and diabetes mellitus [8]. While imaging modalities like USG suggested a benign lesion, MRI played a pivotal role in delineating the extent of the tumor and its relationship with adjacent structures [9]. Differential diagnoses of the malignant mesenchymal tumor include low-grade fibromyxoid sarcomas, sclerosing epithelioid fibrosarcomas, fibrosarcomatous dermatofibrosarcoma protuberans, and synovial sarcomas [9].

Histopathological confirmation through skin biopsy was crucial in establishing the diagnosis of malignant mesenchymal tumor, highlighting the significance of tissue sampling in guiding treatment decisions [10]. The presence of comorbidities such as hypertension and diabetes mellitus underscores the importance of a multidisciplinary approach, as these conditions may influence treatment selection, perioperative management, and overall prognosis [11]. The management of STS often involves a combination of surgery, radiotherapy, and chemotherapy tailored to tumor characteristics and patient factors [12]. In this case, referral to the oncology department for further treatment emphasizes the need for specialized care and expertise in managing rare malignancies like STS. While diagnostic and therapeutic modalities advancements have improved outcomes for patients with STS, challenges remain in optimizing treatment strategies and prognostication. Tumor histology, grade, size, and patient-related variables influence treatment decisions and survival [13].

## Conclusions

In conclusion, this case report of a malignant mesenchymal tumor presenting as an anterior thigh swelling emphasizes the critical role of a systematic diagnostic approach and multidisciplinary management in STS. Despite initial diagnostic challenges and the benign appearance on imaging, histopathological confirmation through skin biopsy was essential for accurate diagnosis and subsequent treatment planning. The case underscores the importance of considering malignant etiologies in patients with soft tissue swellings, particularly in the context of relevant medical history and comorbidities such as hypertension and diabetes mellitus. Moving forward, continued research efforts to refine diagnostic modalities, elucidate tumor biology, and develop personalized treatment strategies are essential for improving the prognosis of STS patients. Additionally, heightened awareness among healthcare professionals regarding the varied presentations of STS and the importance of early diagnosis is crucial for timely intervention and improved survival rates.
